# Culture and awareness of occupational health risks amongst UK firefighters

**DOI:** 10.1038/s41598-022-24845-8

**Published:** 2023-01-10

**Authors:** Taylor A. M. Wolffe, Louis Turrell, Andrew Robinson, Kathryn Dickens, Anna Clinton, Daniella Maritan-Thomson, Anna A. Stec

**Affiliations:** 1 Centre for Fire and Hazards Sciences, University of Central Lancashire, Preston, Lancashire PR1 2HE UK; 2 Royal Preston Hospital, Lancashire Teaching Hospitals NHS Foundation Trust, Preston, Lancashire PR2 9HT UK

**Keywords:** Cancer prevention, Environmental sciences, Risk factors

## Abstract

Firefighters are exposed to toxic chemicals not only from the fire incidents they attend, but also from their contaminated station and/or personal protective equipment (PPE). Little is currently known about firefighters’ awareness, attitudes, and behaviours towards contaminants which was assessed in the UK firefighter contamination survey. Results revealed that lack of training on fire effluents and their health outcomes are strongly associated with increased fire smoke/contaminant exposure. Notably, untrained firefighters were at least twice as likely to: never clean personal protective equipment (PPE) (Crude Odds Ratio, OR 2.0, 1.5–2.7), infrequently send their PPE for professional cleaning (OR 2.0, 1.6–2.4), remain in the workwear (t-shirt etc.) worn while attending a fire incident (OR up to 3.6, 2.3–5.6), and indicate that cleaning at fire stations is not taken seriously (OR 2.4, 2.2–2.6). Firefighters personally viewing contamination as a “badge of honour” (BoH) were at least twice as likely to: remain in contaminated PPE after fire incidents (OR 2.3, 1.4–3.9), eat with sooty hands (OR 2.2, 1.9–2.5), notice soot in the nose/throat (OR 3.7, 2.7–5.2), and smell fire smoke on the body for more than a day after incidents (OR 2.0, 1.6–2.4). They were also more likely to indicate that cleaning at fire stations is not taken seriously (OR 2.5, 2.2–2.9) and that fire stations smell of smoke always/most of the time (OR 2.3, 2.0–2.6). Strong links were also found between belief in the BoH and never cleaning PPE (OR 1.9, 1.4–2.7), and eating while wearing contaminated PPE (OR 1.8, 1.5–2.2).

## Introduction

Many fire contaminants are associated with chronic health outcomes such as cancer^1–3^. Carcinogens have been identified on firefighters’ PPE, equipment, fire station surfaces etc^1,4^. While research has begun to characterise firefighters’ cancers and diseases in relation to their occupational exposures, little is currently known about firefighters’ awareness, attitudes, and behaviours towards contaminants.

When firefighters are subjected to repeated similar experiences (such as fire attendances), over time, they may pay less attention or take less caution over their exposure to toxic substances^5–7^. This puts them at risk of gradually resuming bad habits and routines, leading to illness or accidents^6,8^.

The “badge of honour” (BoH) is an attitude sometimes upheld by firefighters; whereby heavily contaminated personal protective equipment (PPE) is perceived as a mark of prestige^9^. Visibly contaminated PPE provides a performative means of earning the respect of esteemed colleagues, and/or meeting the public’s expectations of firefighters as heroic. BoH and similar attitudes therefore have the propensity to not only increase the wearer’s exposure to fire toxins, but also that of his/her colleagues/family.

This manuscript discusses the influence of cultural beliefs such as the BoH on firefighters’ exposure to contaminants and engagement in decontamination practices. The prevalence of training provision, its potential relationship to BoH belief, and its success at promoting engagement in decontamination practices is also assessed and presented.

## Methods

The methods used to conduct the survey and analyse its results are detailed in Wolffe et al.^10^. Ethical approval for the survey was granted by the University of Central Lancashire Ethics Committee, and all analyses were conducted in accordance with relevant guidelines and regulations. The survey consisted of 64 questions (Supplemental File S1) covering several key topics, including demographics, PPE (provision, maintenance, storage, fit, decontamination practices etc.)^10^, health^11^, and attitudes/awareness and training.

All currently serving (i.e. excluding retired) UK firefighters were eligible to take part in the survey and were recruited to participate via email through the Fire Brigades Union (whose members make up approximately 75% of the UK’s total firefighting workforce^10,12^). Firefighters were invited to anonymously complete the survey online. Informed consent was obtained from all participants.

Belief in the BoH was cross tabulated with several key variables that represented behaviour or practice linked to contaminant exposure (e.g. eating while wearing PPE is a practice/behaviour which acts as a proxy for contaminant ingestion). The training status of firefighters (i.e. whether firefighters had received training on fire contaminants and their health effects) was similarly cross tabulated with behaviour/practice variables. In both instances, BoH/training status was treated as the explanatory (independent) variable, and the practice/behaviour of interest treated as the response (dependent) variable. Thus, the proportion of firefighters in each BoH/training status category who engage in a particular behaviour/practice is presented. An exception is that of the demographics section, which explores the type of firefighter who believes in the BoH/has not received training (i.e. presents BoH/training status as the dependent variable and demographics as independent variables).

Crude Odds Ratio (OR) with 95% confidence intervals were used to assess the statistical significance of differences between the proportion of firefighters who believe in the BoH/have not received training and engage in behaviours/practice which proxy contaminant exposure. Explanatory and response variables were generally collapsed into two categories for each analysis (see Supplemental File S1). A lack of answer or answers of “prefer not to say” were excluded from analyses.

The chi-squared test for trend and z-score test for difference in proportions were also used to assess the statistical significance of apparent trends/findings.

Free-text responses were manually coded by a single author according to an iterative list of the most commonly occurring themes.

## Results and analysis

The responses of a total of 10,649 firefighters were included for analysis, representing approximately 24% of the UK’s total firefighter workforce^10^. Participant demographics were analysed in Wolffe et al.^10^, and were not found to significantly differ from the English firefighter population^13^ with respect to sex (p > 0.05), but appeared to under-represent younger age categories, retained firefighters, and those belonging to an ethnic minority (p < 0.05).

**Figure 1:**
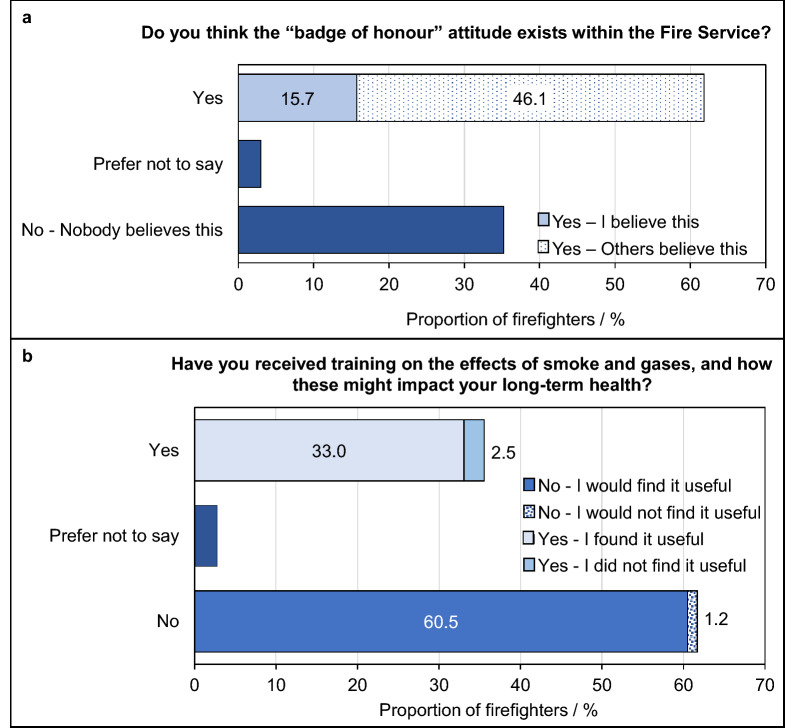
**Training on fire contaminants and their associated health outcomes, and belief in the BoH attitude in the UK Fire and Rescue Service**. Proportion of total surveyed firefighters who (**A**) hold/do not hold the BoH attitude, and (**B**) who have/have not received training on the health effects of fire contaminant exposure.

Approximately sixteen percent of firefighters indicated that they believe visible signs of contamination is a BoH, while a further 46% indicated that they believe others hold this attitude (Fig. 1A).

The majority of firefighters (62%) had not received any training on the health effects of contaminant exposure (Fig. 1B). Of those that had received training, 93% found this training to be useful. Those that had not received training were also receptive to the idea, with 98% indicating that they would find training valuable.

Lack of training only slightly increased the odds ratios for firefighters indicating that they or others believed in the BoH (ORs 1.1, 1.0–1.2, for personal belief in the BoH and 1.1, 1.0–1.2 for others belief). However, this increase was not statistically significant for personal belief in the badge of honour.

### Demographics

The Fire and Rescue Services in the United Kingdom are divided into England, Wales, Scotland and Northern Ireland, and each operates under separate legislative and administrative arrangements^14^. Participant demographics were compared to English FRS statistics^13^ and presented in Wolffe et al.^10^. Detailed analysis of the geographic distribution of belief in the BoH/lack of training can be found in Supplemental File S2.

**Figure 2:**
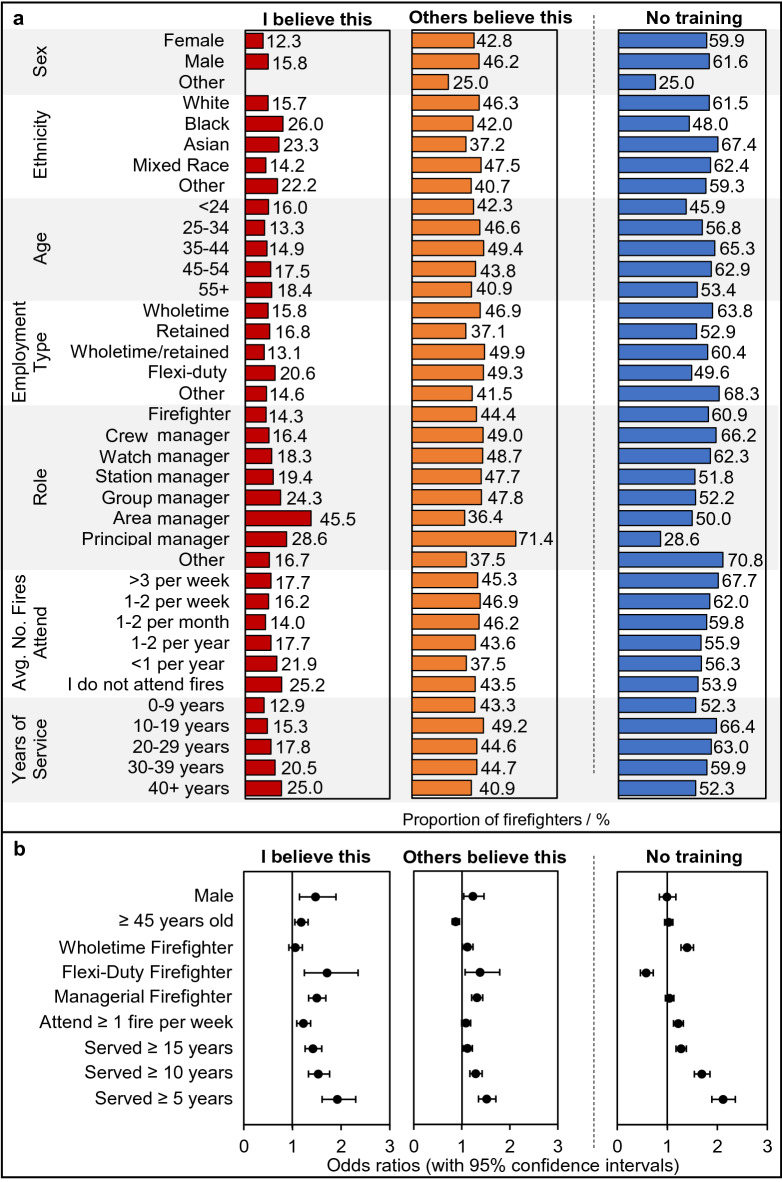
**Firefighter demographics and belief in the BoH, and training on fire contaminants and their associated health outcomes**. (**A**) The proportion of total surveyed firefighters in each demographic category who personally believe in the BoH, who feel others believe in the BoH, and who have not received any training on the health effects of smoke/contaminant exposure. (**B**) Crude odds ratios (with 95% confidence intervals) for belief in the BoH attitude/lack of training and demographics. Note that the small sample size for certain demographic groups (e.g. sex = Other, where n = 4), mean that the above proportions should be interpreted cautiously. Participant demographics are summarised in Wolffe et al.^10^.

The proportion of firefighters indicating personal belief in the BoH generally increased, and was significantly associated with age (p < 0.05), seniority of role (p < 0.05), and years of service (p < 0.05) (Fig. 2). Managerial firefighters (i.e. crew, watch, station, group, area, and principal managers) were 1.5 times more likely to indicate personal belief in the BoH compared to non-managerial firefighters (OR 1.5, 1.3–1.7). Older firefighters (i.e. those 45 or older) were slightly more likely to indicate personal belief in the BoH compared to younger firefighters (OR 1.2, 1.0–1.3).

Belief in the BoH was significantly more likely in firefighters who had served for more than 5, 10, or 15 years (OR 1.9, 1.6–2.3 for 5+, OR 1.5, 1.3–1.8 for 10+, and OR 1.4, 1.3–1.6 for 15+) when compared to firefighters who had served less than 5, 10 or 15 years respectively. In other words, the association between length of service and belief in the BoH was not diluted by regrouping firefighters into wider length of service categories; indicating that belief in the BoH is spread among firefighters who have served for shorter (5+/10+) and longer (15+) lengths of time. Supported by free text analysis, this indicates that belief in the BoH has only very recently begun to wane, i.e. within the last 0–4 years prior to the survey.

Interestingly, the proportion of firefighters personally believing in the BoH was greatest for those that attend fires on the most and least frequent bases, dipping for firefighters who fall between the two extremes (Fig. 2, although small sample sizes at either extreme warrant caution). Fire attendance frequency was found to be significantly associated with personal belief in the badge of honour (p < 0.05), with firefighters attending fires on at least a weekly basis being 1.2 times more likely to report belief in this attitude than those attending fires less frequently (OR 1.2, 1.1–1.4).

Trends in the number of firefighters who believed that others held the BoH attitude were less clear, with roughly equal proportions of each demographic category indicating that others held the attitude (Fig. 2), and no significant associations found for fire attendance frequency (p > 0.05), or years of service (p > 0.05). However age (p < 0.05) and seniority of role (p < 0.05) were still found to be significantly associated with others’ belief in the badge of honour.

Fire attendance frequency was significantly (p < 0.05) and positively correlated with a lack of training (Fig. 2). Firefighters attending fires at least once a week were 1.2 times more likely to have had no training on fire contaminants compared to those attending fires less frequently (OR 1.2, 1.1–1.3).

Years of service was also found to be significantly associated with lack of training (p < 0.05). Firefighters who had served 15 years or longer were found to be slightly but significantly more likely to indicate that they had not received training compared to those that had served less time (OR 1.3, 1.2–1.4). This was similarly found for firefighters who had served 5 + years (OR 2.1, 1.9–2.4) or 10+  years (OR 1.7, 1.5–1.9). Free text answers also indicated that training on contaminants may only have been introduced in the last 0–4 years before the survey.

A significant association was additionally found between lack of training and age (p < 0.05), but not between lack of training and seniority of role (p > 0.05).

### Personal contamination during/after fire incidents

Crude odds ratios for firefighters attending incidents without respiratory protective equipment (RPE) were significantly increased for both belief in the BoH and lack of training on fire contaminants (Fig. 4). Firefighters who personally believed in the BoH, or who had not received training, were both 1.7 times more likely to attend incidents without RPE compared to those who felt nobody believed in the BoH, or who had received training (OR 1.7, 1.5–1.2 and OR 1.7, 1.5–1.9 respectively). Those who felt others believed in the BoH were still more likely to attend incidents without RPE, albeit to a lesser extent than those who personally believed the attitude (OR 1.4, 1.3–1.6).

Culture, awareness and training featured strongly among firefighters’ reasons for attending fires without RPE. Free text responses revealed that firefighters were left to make their own judgements on the use of RPE (14% firefighters providing free text), or felt pressured by peers to attend fires without RPE (7% firefighters providing free text).

**Figure 3:**
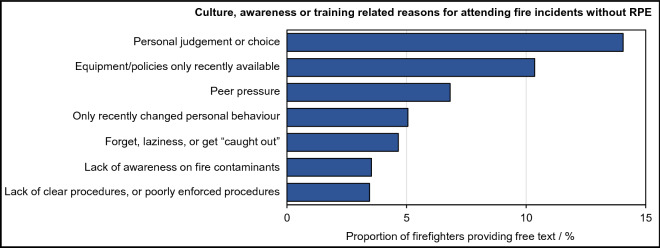
**Culture, awareness and training and attending fires without RPE**. Cultural/awareness related reasons for firefighters’ attending fire incidents without respiratory protective equipment, coded from free text. Note that a single firefighter’s free-text answer may have contained several sentiments belonging to several different themes.

Around 4% of firefighters providing free text reasons for attending fire incidents without RPE specifically cited a lack of procedures or poorly enforced procedures concerning when to use RPE, or which RPE was most appropriate to use (Fig. 3). It should be noted that a lack of/poorly enforced procedures are likely to facilitate firefighters being left to make their own judgements on the use of RPE. Thus, while firefighters may not always have mentioned both issues in their free text responses, they are likely to be closely linked.

**Figure 4:**
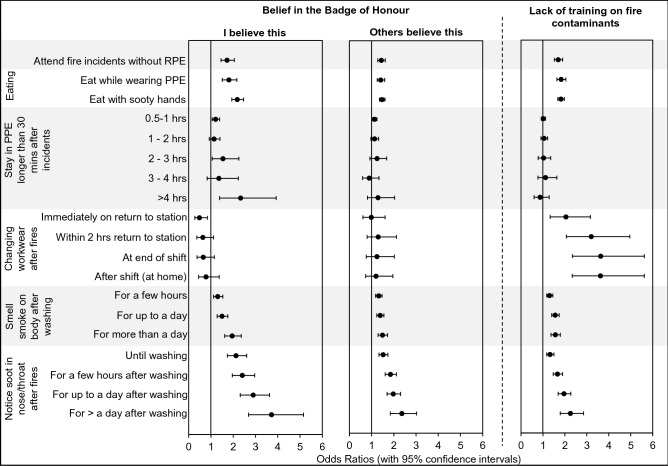
**Crude odds ratios for engaging in behaviours/practices that lead to personal contamination during/after fire incidents for firefighters who believe in the BoH, or who have not received training on fire contaminants and their associated health outcomes**.

Firefighters who believe in the BoH were significantly more likely to eat while wearing PPE (OR 1.8, 1.5–2.2 for personal, and OR 1.4, 1.3–1.6 for others belief in the BoH), Fig. 4. Similarly, a significantly increased odds ratio for eating with sooty hands was found for personal/others belief in the BoH (OR 2.2, 1.9–2.5 for personal, and OR 1.5, 1.4–1.6 for others’ belief in the BoH, respectively). A statistically significant increased odds ratio was also found for staying in PPE for an extended amount of time after attending fire incidents among those who personally believed in the BoH (OR 2.3, 1.4–3.9 for > 4 h). This was not found for firefighters who claimed that others believed in the BoH (Fig. 4).

BoH attitude did not appear to influence the odds ratios for firefighters remaining in their workwear after attending a fire incident (Fig. 4).

Except for remaining in PPE for an extended amount of time after a fire incident, a lack of training was associated with a significantly increased odds ratio for every measure of personal contamination assessed in the survey, i.e. attending fire incidents without wearing RPE (OR 1.7, 1.5–1.9); consuming food with sooty hands (OR 1.8, 1.7–2.0), or while wearing PPE (OR 1.8, 1.6–2.0); smelling smoke on the body after fire incidents (OR 1.3, 1.2–1.4 for a few hours after washing, OR 1.6, 1.4–1.7 for up to a day after, OR 1.6, 1.4–1.8 for more than a day after); and noticing soot in the nose/throat after fire incidents (OR 1.3, 1.2–1.5 until washing, OR 1.7, 1.5–1.9 for a few hours after washing, OR 2.0, 1.7–2.3 for up to a day after, OR 2.3, 1.8–2.8 for more than a day after). Most notably, firefighters who did not receive training were up to 3.6 times more likely to remain in their workwear for an extended amount of time after attending an incident (OR 3.6, 2.3–5.6 for changing workwear at home after finishing a shift, Fig. 4).

For firefighters who believed in the BoH, the odds ratios for both continuing to smell smoke on the body, and for noticing soot in the nose/throat, increased as the length of time after washing on return from an incident increased (Fig. 4). When compared to those who felt nobody believed in the BoH, these firefighters were 2.0 times more likely to smell smoke on their bodies (OR 2.0, 1.6–2.4), and 3.7 times more likely to notice soot in their nose/throat, for more than a day after washing on return from a fire incident (OR 3.7, 2.7–5.2, Fig. 4).These trends were mirrored by firefighters who felt that others believed in the BoH.

Compared to those who felt nobody believed in the BoH, a consistently larger proportion of firefighters who believed in the BoH, or felt their colleagues did, rated face (49% and 38% vs. 36%), neck (32% and 26% vs. 23%), hair (40% and 36% vs. 32%), and hands (54% and 50% vs. 45%) as feeling highly contaminated after fire incidents (Supplemental File S2, Fig. S3).

As expected, a larger proportion of these firefighters also consistently rated hair (65% and 60% vs. 57%) and hands (49% and 43% vs. 38%) as smelling strongly of smoke (Supplemental File S2, Fig. S4). These trends were mirrored for firefighters who had not received training (Supplemental File S2, Figs. S3, S4).

### Personal protective equipment

**Figure 5:**
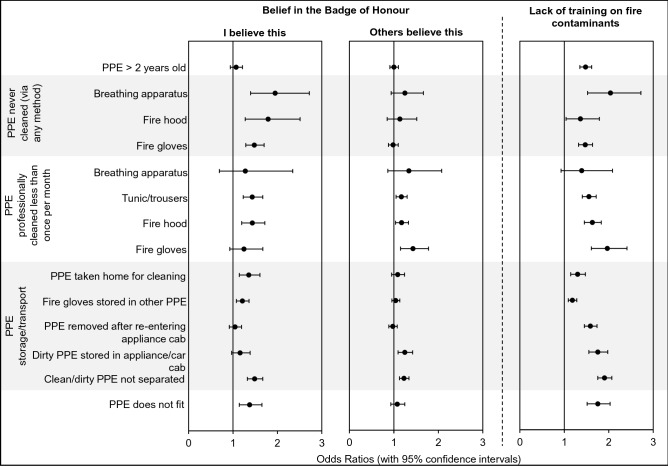
**Belief in the BoH/lack of training and crude odds ratios for engaging in negative PPE storage or cleaning practices**. Although the survey asked firefighters whether they sent breathing apparatus and fire gloves for professional cleaning—it should be noted that sending these items for external, professional cleaning is not (currently) standard practice^10^.

A lack of training and belief in the BoH, were both significantly associated with several negative behaviours/practices concerning the storage and cleaning of PPE (Fig. 5), i.e. those that would put firefighters at a potentially increased risk of contaminant exposure, or would lead to harmful cross contamination.

Personal belief in the BoH significantly increased the odds ratios for firefighters never cleaning their PPE (Fig. 5) by as much as 1.9 times (BA never cleaned, OR 1.9, 1.4–2.7). Similarly, belief in the BoH was associated with failing to send PPE for professional cleaning on a regular basis (i.e. sending PPE for professional cleaning less than once per month), although this was only found to be significant for tunics/trousers (OR 1.4, 1.2–1.7) and fire hoods (OR 1.4, 1.2–1.7). Firefighters were also significantly more likely to wear ill-fitting PPE (OR 1.4, 1.1–1.7), and to engage in PPE storage/transport practices known to contribute to cross-contamination if they believed in the BoH. This included taking PPE home to clean (OR 1.4, 1.1–1.6), storing fire gloves within other items of PPE e.g. helmets, boots, tunic pockets etc. (OR 1.2, 1.1–1.4), and failing to store clean/dirty PPE separately (OR 1.5, 1.3–1.7).

Statistical significance was also found for the increased odds ratio associated with firefighters who indicated that their colleagues believed in the BoH sending their PPE for cleaning on an infrequent basis (OR 1.4, 1.1–1.8 for fire gloves, OR 1.2, 1.1–1.0–1.3 for fire hoods, OR 1.2, 1.1–1.3 for tunic/trousers). In addition, a significantly increased odds ratio was found for these firefighters failing to store clean/dirty PPE separately (OR 1.2, 1.1–1.3) and storing used/dirty PPE in the appliance/car cab on return from an incident (OR 1.2, 1.1–1.4). Interestingly, there was no significant association between firefighters who indicated their colleagues believed in the BoH and never cleaning their PPE (Fig. 5).

Lack of training was also associated with a significantly increased odds ratios for all negative PPE storage/cleaning practices assessed in the survey. An exception was for failing to send breathing apparatus (BA) for regular professional cleaning. However, it is not standard practice to send this item for external, professional cleaning (Fig. 5). Notably, a lack of training doubled the odds ratio for firefighters *never* cleaning BA (professionally or otherwise, OR 2.0, 1.5–2.7), sending fire gloves for professional cleaning infrequently/never (OR 2.0, 1.6–2.4), storing dirty/used PPE in the appliance or vehicle cab on return from incidents (OR 1.8, 1.6–2.0), storing clean and dirty PPE together (i.e. not separately, OR 1.9, 1.8–2.1) and wearing PPE that does not fit well (OR 1.8, 1.5–2.0).

When asked what firefighters’ main reasons for cleaning their PPE were, roughly equal proportions of those from each BoH belief category (i.e. I believe this, others believe this, nobody believes this) selected each of the answer options (Supplementary File S2, Fig. S2). The exceptions were for “I send it after every incident” and “It is damaged”—where a smaller proportion of those who believe in the BoH indicated that these were reasons they would send their PPE for cleaning compared to those who felt nobody believed in the BoH.

A similar trend was observed for training status—whereby a smaller proportion of those who had not received training indicated that they would send their PPE for cleaning after every incident compared to those who had received training (Supplementary File S2, Fig. S2).

### Workplace contamination

A statistically significantly larger proportion of firefighters personally holding the BoH attitude indicated that their station had no separate clean/dirty areas (28%) for PPE compared to firefighters who felt others believed in the BoH attitude (22%) (p < 0.05), and compared to firefighters who felt that nobody believed in the BoH attitude (20%) (p < 0.05).

**Figure 6:**
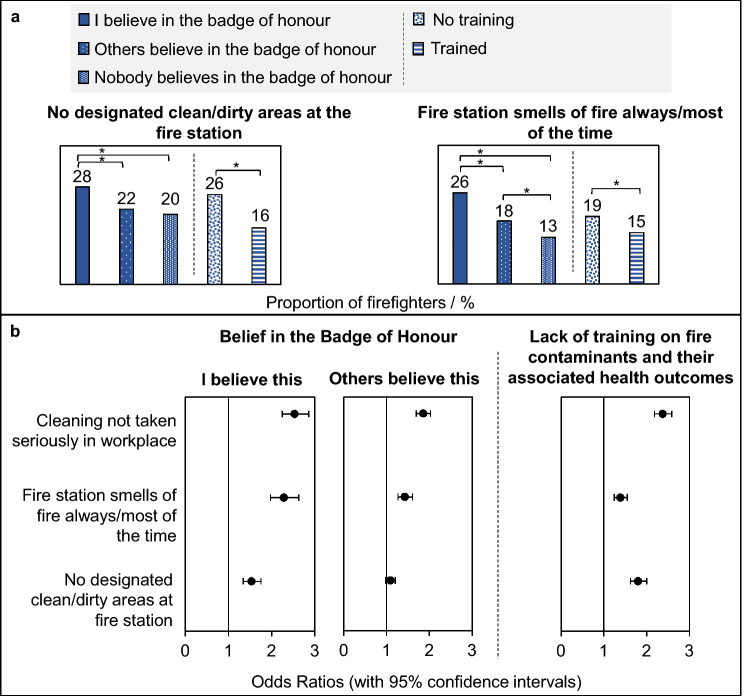
**Belief in the BoH/lack of training and workplace contamination**. (**A**) The proportion of firefighters, and (**B**) the crude odds ratios, for firefighters in each BoH belief/training status category whose workplaces have no designated clean/dirty areas or smell of fire always/most of the time. *Indicates a p-value of < 0.05 for the z-score test for difference in proportions.

Similarly, a significantly larger proportion of firefighters personally holding the BoH attitude indicated that their workplace smelt of fire/smoke always/most of the time (26%) compared to those who felt that others believed in the BoH (20%) (p < 0.05), and compared to those who felt nobody believed in the BoH (13%) (p < 0.05), Fig. 6.

These findings were mirrored for firefighters who had not received training (compared to firefighters who had received training, Fig. 6), i.e. p < 0.05 for the difference in proportions between trained and untrained firefighters whose workplace smells of fire always/most of the time, or whose workplaces have no designated clean/dirty areas.

Firefighters who personally believed in the BoH were found to have odds ratios for smelling fire in the station always/most of the time which were more than twice that of firefighters who felt that nobody believes in the BoH, Fig. 6 (OR 2.3, 2.0–2.6). Firefighters who did not receive any training on fire contaminants were 1.8 times more likely to work in a station without dedicated clean/dirty areas compared to those that had received such training (OR 1.8, 1.6–2.0).

It was also found that firefighters who personally believed in the BoH, or who had not received training on fire contaminants, were over twice as likely to indicate that cleaning was not taken seriously at their workplace compared to those who felt nobody believed in the BoH/had received training (OR 2.5, 2.2–2.9 and OR 2.4, 2.2–2.6, respectively). Firefighters who felt that their colleagues believed in the BoH were also significantly more likely to indicate that cleaning was not taken seriously at their workplace (OR 1.9, 1.7–2.0), albeit to a lesser extent than those who personally held the attitude.

Firefighters were also given the opportunity to provide their thoughts and feelings on the subject of fire contamination at their workplace, via free-text. Around 38% of all firefighters (n = 4072) provided free text responses to this prompt, covering a variety of issues including training provision and culture/awareness. Most notably, 13% of firefighters who answered the question indicated discrepant attitudes toward cleaning and contamination at their workplace, (e.g. “Some people take it seriously, but others don’t”). Approximately, 1% specifically mentioned the BoH, with similarly discrepant views on the demographic of firefighters who hold the attitude (e.g., “Cleaning is not taken seriously by some, mostly new personnel and some experienced personnel. I feel some think it’s a badge of honour to be covered in soot”).

Training also received mention among free text responses. Just 0.2% of firefighters left positive sentiments towards training/awareness. By contrast, 2.4% of firefighters providing free text felt that training/awareness was inadequate, or that further training was required.

Encouragingly, around 9% of firefighters providing free text felt that attitudes/awareness were improving (e.g. “It is getting better with the more we learn”). Many of these firefighters indicated that training had very recently been introduced, or that awareness was only recently increasing. However, many indicated that there was still much work needed to protect firefighters from fire contaminant exposure, or that the pace of change was still slow, e.g. “I can see the new research and findings taking a positive effect on station but it’s not as fast a change as perhaps it needs to be”, “it is only within the last 6 months that it has reached the radar…still a long way to go”).

## Discussion

### Procedures and regulations versus awareness and habits

The results of the survey uncovered considerable variation in the decontamination practices firefighters engage in. Firefighters from the same demographic or geographic region indicated different levels of compliance with decontamination procedures, and/or varied training provision. For example, firefighters within the same geographical regions indicated storing clean and dirty PPE separately or not, or that their workplaces have/have not designated clean/dirty areas (Supplemental File S2), suggesting a potential lack of standardisation of decontamination policies and/or a lack of compliance with such policies in the UK Fire and Rescue Services. This was similarly found in earlier work, when reviewing UK FRS decontamination policies^15^, and was a sentiment which was echoed in firefighters’ free text responses to the current survey, e.g. “My Watch Manager on my on-call station doesn’t follow the policy all the time so is rarely cleaned and decontaminated after incidents”, “I wrote an SOP [standard operating procedure] relating to decontamination and kit cleaning after attending a course. All agreed, but it was never followed or taken seriously by management”, “cleaning is taken seriously by most but not all. Very little from headquarters to reiterate what must be done”.

Without such standardisation, several firefighters indicated that they felt judgements on contaminants were left to personal choice, e.g. “PPE cleanliness is generally down to personal choice I find…”, “There is no clear guidelines. It seems to be personal preference”, “…I believe it should be enforced strictly as it is normally down to personal preference”. As well as practicality and resources, firefighters indicated that these judgements were sometimes based on a personal assessment of risk, e.g. reasons given for attending fire incidents without RPE such as “own risk assessment”, “I believe I risk assess the appropriateness for the need to wear PPE. Many occasions when I attend I am for example up wind out of smoke, if I decide to enter a smoke area or cold fire scene I may use the PPE or RPE”, “Assess where the smoke is, and what it is, and if it affects me I will wear one [RPE]”. This is particularly detrimental for firefighters who are not fully aware of the dangers of contaminant exposure and/or the routes via which firefighters are exposed to chronic, low doses of contaminants.

“Habit” was another reason commonly given for engaging in negative contaminant exposure practices e.g., attending incidents without RPE. While some firefighters left free text responses which implied they knew these habits may have negative effects on their health (“sometimes I don’t think I need it [RPE], but probably should”) several also indicated that they had changed their behaviour after gaining knowledge on fire contaminants and their health effects, e.g. “I have attended incidents for years without being aware of the risks especially post fire. Things are changing now but this needs to become embedded as normal practice”, “never used to [wear RPE] but am more mindful now”.

Thus, providing training as an intervention which increases awareness of the dangers of contaminant exposure, in combination with stricter regulations and better standardised decontamination practices, may be required in order to protect firefighters from exposure to harmful fire contaminants.

### Provision of training on fire contaminants and their associated health outcomes

The majority (62%) of firefighters in the UK Fire and Rescue Service have not received any training on fire contaminants and their health effects. This is particularly concerning given that a lack of training increased the odds ratios for firefighters engaging in a variety of behaviours/practices which not only increase their exposure to harmful contaminants, but also increase their likelihood of developing cancer^11^. These behaviours/practices either expose firefighters directly (e.g. eating while wearing PPE, attending fires without RPE etc.)—or indirectly through cross contamination (e.g. failing to store clean and dirty PPE separately).

Also of concern is the finding that firefighters most at risk of exposure to contaminants are also most likely to have received no training on the health effects of the contaminants they are exposed to, i.e. those attending fires on at least a weekly basis were 1.2 times more likely to have received no training compared to those attending incidents less frequently (OR 1.2, 1.1–1.3). A significantly larger proportion of firefighters attending fires on a weekly basis were found to have served 15 years or longer compared to those attending fires less frequently (p < 0.05). Further breakdown of these trends finds firefighters who had served more than 5 years still had an increased odds ratio for having received no training (OR 2.1, 1.9–2.4). Thus, training may only have been introduced very recently (i.e. within 0–4 years before the survey was conducted), which is supported by free text responses, e.g. “we have only just recently started to adhere to a clean cab policy following on from evidence coming out about the risk of cancers to firefighters”, “in the past year we understand the risks from contaminants so take greater care for cleaning equipment. For the previous two decades this was just a cursory clean, basically as long as soot didn’t come off on your hands then the equipment was fine”.

This also implies that there is a legacy group of firefighters who never received training on the health effects of contaminant exposure, and who continue to be overlooked in terms of training provision, even if new recruits have since received such training.

Compared to more obvious or direct indicators of contamination (e.g. workplace smelling of smoke, spending an extended amount of time in PPE after incidents etc.), a lack of training appeared to be most strongly associated with more subtle or indirect proxies of contamination (compared to belief in the BoH). For example, the greatest odds ratios for working in a fire station without designated clean/dirty areas was found for firefighters who had not received training (Fig. 6, OR 1.8, 1.6–2.0). Similarly, firefighters without training were most likely to stay in their workwear (i.e. the clothes worn underneath PPE) for an extended amount of time after attending fire incidents (OR 3.6, 2.3–5.6 for changing workwear at home after the end of a shift), or to engage in PPE transport/storage practices known to cause cross contamination, e.g. de-robing dirty PPE after re-entering the appliance cab (Fig. 4, OR 1.6, 1.5–1.7).

These more subtle measures of exposure to fire contaminants are perhaps easily overlooked, leading to chronic exposure. Exposure to (even very low doses of) contaminants which are carcinogenic or endocrine disrupting (e.g. polycyclic aromatic hydrocarbons, polybrominated diphenyl ethers, etc.) can illicit health conditions with long latent periods such as cancer. Further, chronic exposure to such chemicals makes these health outcomes increasingly likely. Given the above discussed results, it can be assumed that training increases awareness of the health effects associated with chronic, low-dose exposures—and promotes the uptake of policies/procedures which target such exposures.

Having said that, considerable proportions of firefighters who did receive training still indicated engagement in negative behaviours/practices regarding fire contamination. For example, 80% of trained firefighters indicated that they would attend fire incidents without RPE, while 81% indicated that they would eat while wearing PPE. In some instances, this may plausibly be due to discrepant training status among firefighters working in the same station. For example, new recruits who have received training may begin working in stations that are staffed by firefighters who have not received such training, and who may therefore unknowingly perpetuate negative contamination practices.

Firefighters who *have* received training may also be subject to “habitual blindness”^7^—where the frequency with which they have historically engaged in negative contamination practices desensitises them to the potential dangers of such practices, even if previously educated on the health implications. Thus, regular training updated with the latest scientific evidence on fire contaminants, their exposure routes and health outcomes may be required in order to tackle such habitual blindness.

Alternatively, continued engagement in such practices may be more significantly influenced by cultural factors such as the BoH attitude and similar bravado—reflected in several of the survey’s free text responses. This was also found to be relevant for a surveyed population of US firefighters, where cultural influences such as peer pressure were found to be a major influence on firefighters’ choice to use PPE^16^.

In fact, several studies in non-firefighting occupational groups similarly find a disconnect between high levels of safety knowledge and the use of PPE in practice, demonstrating the importance of exploring cultural modifiers of safety behaviour^17,18^.

### Badge of honour

The BoH is an attitude which has historically been strongly associated with the Fire and Rescue Service^9^, and celebrates visibly contaminated PPE as a mark of hard work. Firefighters who personally believe in the BoH had the greatest odds ratios for engaging in practices/behaviours which are linked to PPE contamination, e.g. never cleaning PPE (Fig. 3).

Interestingly, the survey found that those who felt others believed in the BoH (46% of all surveyed firefighters) were also more likely to partake in behaviours/practices that increase the risk of exposure to contaminants, although generally to a lesser extent than those who personally hold the attitude. These findings indicate the potentially considerable influence of peer pressure in the UK Fire and Rescue Service, and/or the need to conform to cultural expectations. Examples of peer pressure modifying firefighters’ behaviour were found in the free text reasons given for attending fire incidents without RPE, e.g. “I do wear it occasionally but when I don’t it’s usually due to nobody else wearing it and an element of peer pressure”, “peer pressure, Bravado from colleagues”, “it is the attitude not to wear it and seen as weak”, “pressure from others—would get some stick”.

Unsurprisingly, firefighters who felt their colleagues believed in the BoH were also more likely to indicate that cleaning was not taken seriously at their workplace (OR 1.9, 1.7–2.0). This illustrates the far-reaching impacts of non-compliance to decontamination practices—as even a single non-compliant colleague has the potential to lead to cross contamination which increases the exposure of complaint colleagues^10^. This may in turn compromise overall compliance, as individuals following decontamination procedures feel their efforts are undone by noncompliant colleagues.

Some research suggests that the BoH is an outdated attitude which is becoming less common in Fire and Rescue Services globally^9^, particularly as awareness of the harmful effects of fire contaminants grows. As such, firefighters personally believing in the BoH were in the minority (16%) in the UK Firefighter Contamination Survey (compared to 46% indicating others believe in the BoH, and 35% indicating nobody believes in it, Fig. 1). Consistent with this research, the proportion of firefighters personally believing in the BoH was positively and significantly correlated with age, years of service, fire attendance frequency, and seniority of role (Fig. 1). Those attending fires on the most and least frequent bases were the demographic categories with the largest proportions of firefighters personally believing in the BoH. This may result from a desensitisation to contaminant exposure for those frequently attending incidents, whereas those attending fires least frequently may feel extra pressure to prove their worth. Alternatively, the interconnected relationship between age, years of service and seniority of role may offer explanation for the latter finding, as more senior ranking managerial firefighters (who may still uphold the outdated BoH attitude) begin attending fewer incidents as their careers progress.

Consistent with the odds ratios calculated for firefighters serving 15+,10+ or 5+ years above, analysis of free text responses concerning PPE/workplace cleaning practices revealed an inconsistent picture of belief in the BoH within the UK Fire and Rescue Service. Some firefighters blamed new recruits for perpetuating the attitude, while others cited older firefighters/management as continuing to uphold the attitude e.g. “younger crew members see a dirty kit as a badge of honour…”, “some new firefighters think it’s cool and good that their kit smells of fire after having a working job and wearing BA. It’s like it’s a status”, “I think among the people with more years in the job the smell after a fire is seen as a badge of honour…”, “getting the old crew to clean is a problem”, “old hands rarely if ever clean kit, let alone send it off to be cleaned professionally. I have routinely been told to take my gloves and flash hood home to clean in the washing machine which I have refused to do so. I am shocked with how some people are so stuck in older times and refuse to look at the evidence-based research…”.

## Limitations and future work

As discussed in previous parts of the UK Firefighter Contamination Survey analysis^10,11,19^, the survey may be subject to participation bias.

In this manuscript, a lack of training on fire contaminants and belief in the BoH were treated as independent variables, and engagement in certain negative contamination behaviours/practices treated as dependent variables. Crude odds ratios were not adjusted for other factors associated with the outcome variables as in  other analyses of the UK Firefighter Contamination Survey^11,19^, due to the large variety of outcomes assessed in this manuscript. While it may have been possible to devise a list of plausible risk factors (e.g. access to changing facilities) for engaging in negative behaviours/practices, the survey did not ask firefighters for these details and therefore cannot account for these plausible justifications. Thus, the OR presented in this manuscript may be an oversimplification of the true relationship between lack of training on fire contaminants or belief in the BoH and engagement in practices which potentially increase contaminant exposure. These crude results may be used to inform future work, whereby a more comprehensive multi-variate model analysis may be used (in conjunction with additional survey questions) to account for the presented (and other) confounding variables.

Some of the negative behaviours/practices assessed in the survey may be beyond the control of firefighters. For example, frequency of sending PPE for professional cleaning may be determined by management, fire station layout may make it impossible to completely separate clean/dirty areas etc. In these circumstances, the contribution of training/BoH belief to firefighters’ engagement in such practices may be overstated.

As previously reported^10^, the negative contamination practices assessed in the survey only represent firefighters’ current activities and do not account for past behaviour.

Similarly, the survey cannot verify the quality or content of the training received by firefighters, or when in their careers this training was received. Thus, training status may be an over-simplified representation of firefighters’ awareness of the health risks associated with contaminant exposure, and the practices which lead to it.

## Conclusions

This manuscript identified belief in the BoH and a lack of training on fire contaminants as risk factors for firefighters engaging in practices which increase their exposure to toxic fire contaminants. At the time the survey was conducted, only 36% of firefighters had received training on fire contaminants and their associated health outcomes. A lack of training increased the odds ratios for firefighters engaging in practices/behaviours which increase exposure to contaminants, particularly those leading to cross-contamination.

Culture also appeared to strongly influence firefighters’ engagement in behaviours/practices which increase contaminant exposure. In particular, those who personally believed in the BoH were significantly more likely to indicate engaging in practices which lead to direct exposure. Those indicating their colleagues believed in the BoH were also more likely to engage in such practices.

Evidence from the UK Firefighter Contamination Survey suggests that awareness and training (and the effective decontamination practices that are associated with it), have very recently been introduced to the UK Fire and Rescue Service. However, given the weak association between training status and personal belief in the BoH, and firefighters’ continued engagement in poor decontamination practices as a result of peer pressure—there is still a need for a more fundamental culture shift in the UK Fire and Rescue Service, in combination with increased training provision.

## Supplementary Information

Supplementary Information 1.

Supplementary Information 2.

## Data Availability

The datasets generated and/or analysed during the current study are available from the corresponding author on reasonable request.
