# Supplementary material for: Culture and awareness of occupational health risks amongst UK firefighters
Source: Sci Rep. 2023 Jan 10;13:97. doi: 10.1038/s41598-022-24845-8 (PMC9832120; doi:10.1038/s41598-022-24845-8)
Supplement: Supplementary file 2 — Supplementary Information 2. [file 41598_2022_24845_MOESM2_ESM.docx]

**Supplemental File S2**

Culture and Awareness of Occupational Health Risks Amongst UK Firefighters

Taylor A. M. Wolffe^1^, Louis Turrell^1,2^, Andrew Robinson^1,2^, Kathryn Dickens^1^, Anna Clinton^1^, Daniella Maritan-Thomson^1^, Anna A. Stec^1,*^

^1^Centre for Fire and Hazards Science, University of Central Lancashire, Preston, PR1 2HE

^2^Royal Preston Hospital, Lancashire Teaching Hospitals NHS Trust, Preston, Lancashire, PR2 9HT, UK

^*^Corresponding author: [aastec@uclan.ac.uk](mailto:aastec@uclan.ac.uk)

**Additional Methodological Detail**

The survey was piloted with a small subset of firefighters, and questions rephrased for clarity according to feedback. Ethical approval for the survey was granted by the University of Central Lancashire Ethics Committee, and all analyses were conducted in accordance with relevant guidelines and regulations.

The survey ran through Jisc software, for a period of 3 months between November 2019 and February 2020. A link to the survey was distributed to participants via email through the Fire Brigades Union (FBU). The survey took approximately 20 minutes to complete and was supported by UK Fire and Rescue Services (FRSs) with respect to allowing firefighters dedicated time within their workday in which to complete it.

Free text answers were manually coded for analysis according to the most commonly appearing themes.

All currently serving UK firefighters were eligible to take part in the survey. Therefore, the first question of the survey, *“Are you currently working as a firefighter in the UK?”*  was used to include/exclude survey responses from analysis. A total of 6 respondents indicated that they were **not** currently working as firefighters in the UK and were thus excluded from further analysis. A further 4 respondents identified themselves as retired in the free text answers they provided to survey questions and were thus excluded. Four hundred and seventy-one respondents left this initial question blank. Due to the nature of recruitment to the survey (i.e. via email to FBU members), these respondents were assumed to be currently serving UK firefighters and included in subsequent analyses. This left a total of 10,649 included respondents. This figure represents approximately 24% of the UK’s total Firefighter workforce.

**Geographic Distribution of Training Provision/Belief in the BoH**
The badge of honour attitude was personally held by 6-25% of respondents in all FRSs except for Guernsey, where 0% of firefighters personally held the attitude (Figure S1). Thirty three to 64% of respondents from a particular FRS indicated that others believed in the badge of honour (except for Guernsey, where 100% of respondents indicated that they thought others believed in it, Figure S1). For training, between 33 and 78% of respondents from a particular FRS indicated that they had not received any training on the health effects of contaminant exposure (Figure S1).

**Figure S1: Geographic distribution of belief in the badge of honour/lack of training.** The percent of total respondents from each FRS (i.e. those who participated in the survey) who believe in the BoH or have not received training on the health effects of contamination exposure.

**PPE**


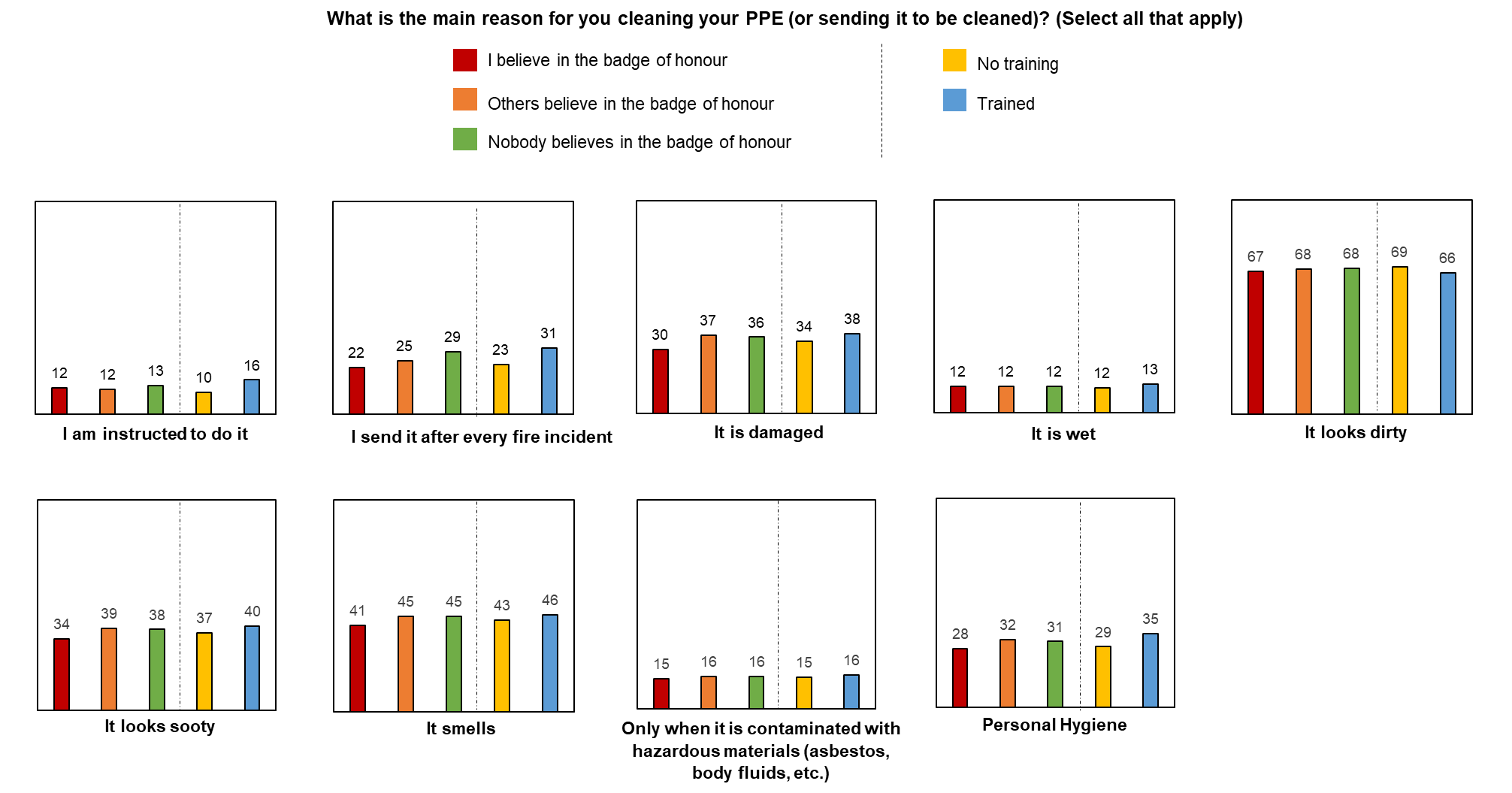


**Figure S2**: **Firefighters’ reasons for cleaning their PPE (selected from a list of options).** The percentage of firefighters from each badge of honour belief category/training status category who selected each of the listed options is presented (e.g. 208 respondents who believe in the BoH selected “I am instructed to do it” out of a total of 1674 respondents who believe in the badge of honour – presented as 12% to aide comparison).

**Personal Contamination**

**
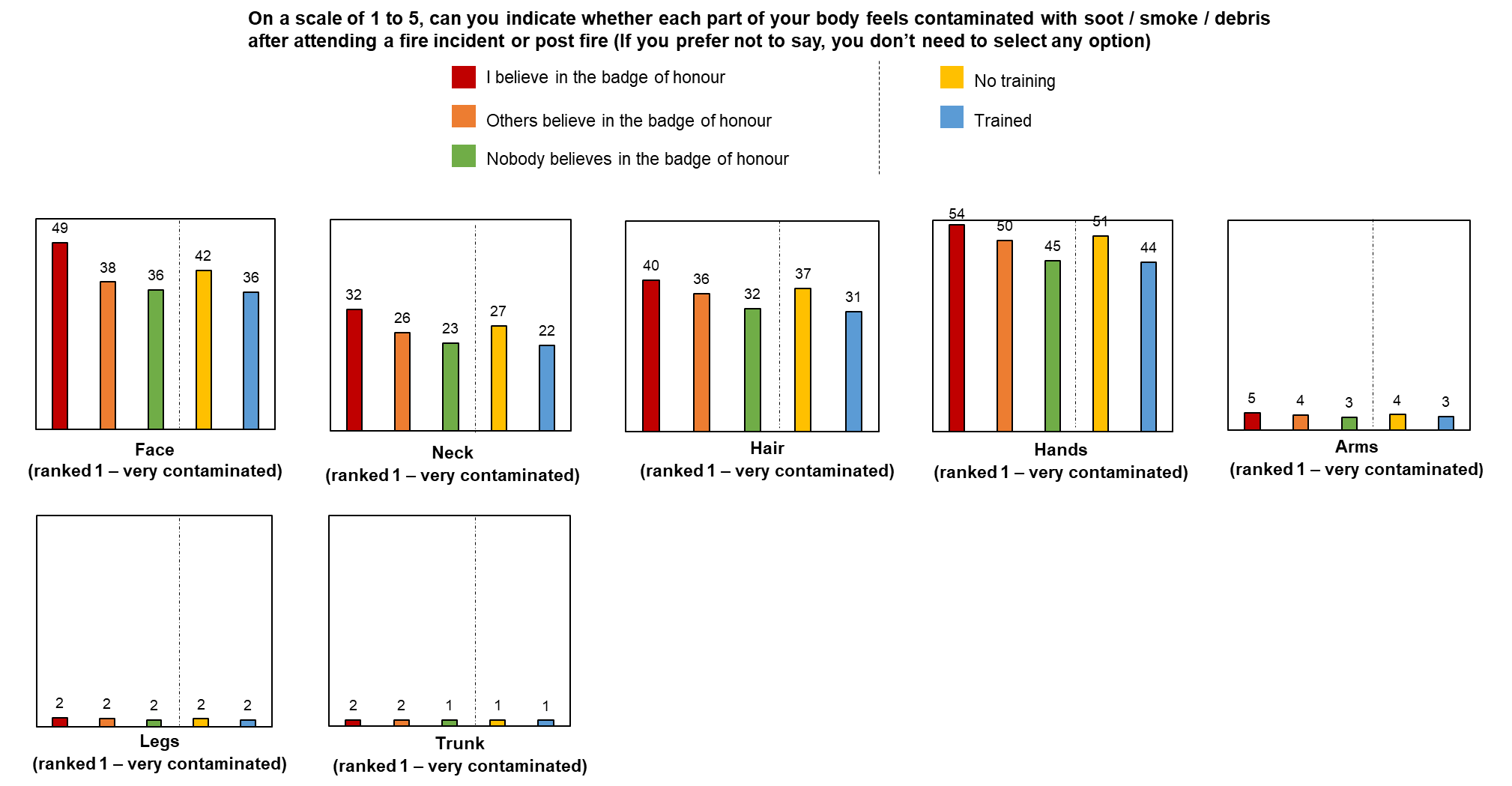
**

**Figure S3**: **Firefighters’ ratings of various body parts feeling very contaminated.** The percentage of firefighters from each badge of honour belief category/training status category who ranked each of the listed body parts as “1 – very contaminated” is presented.

**
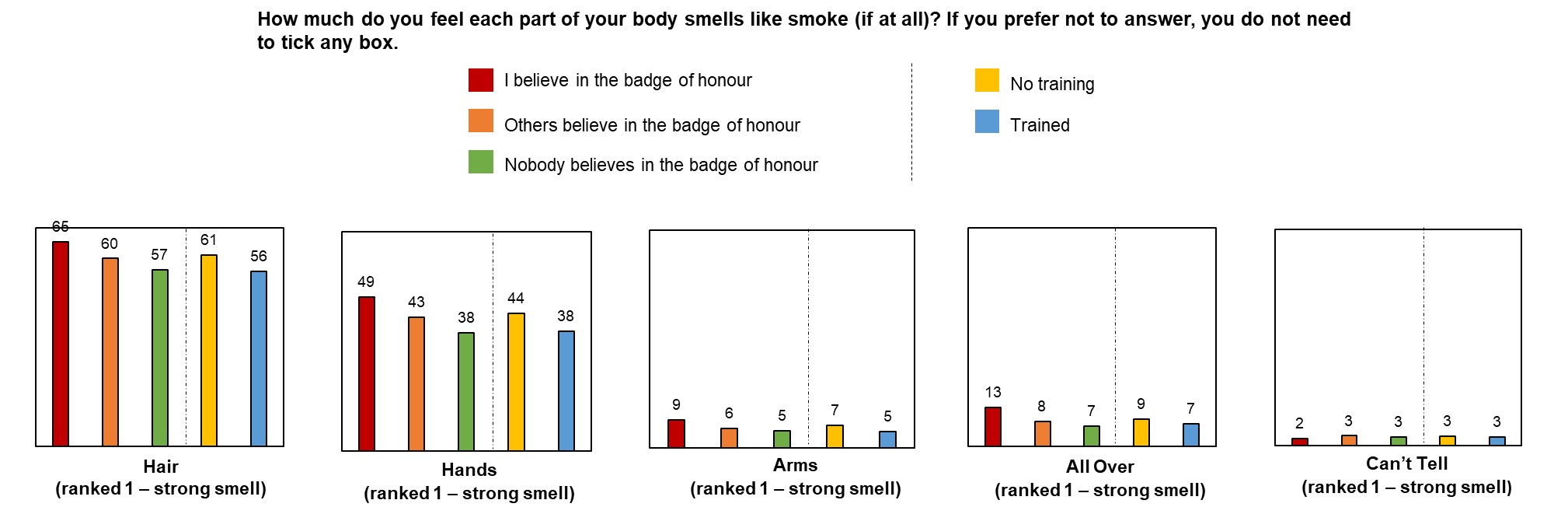
**

**Figure S4: Firefighters’ ratings of various body parts smelling of smoke.** The percentage of firefighters from each badge of honour belief category/training status category who ranked each of the listed body parts as “1 – smells strongly (of smoke)” is presented.

**Incident Contamination**


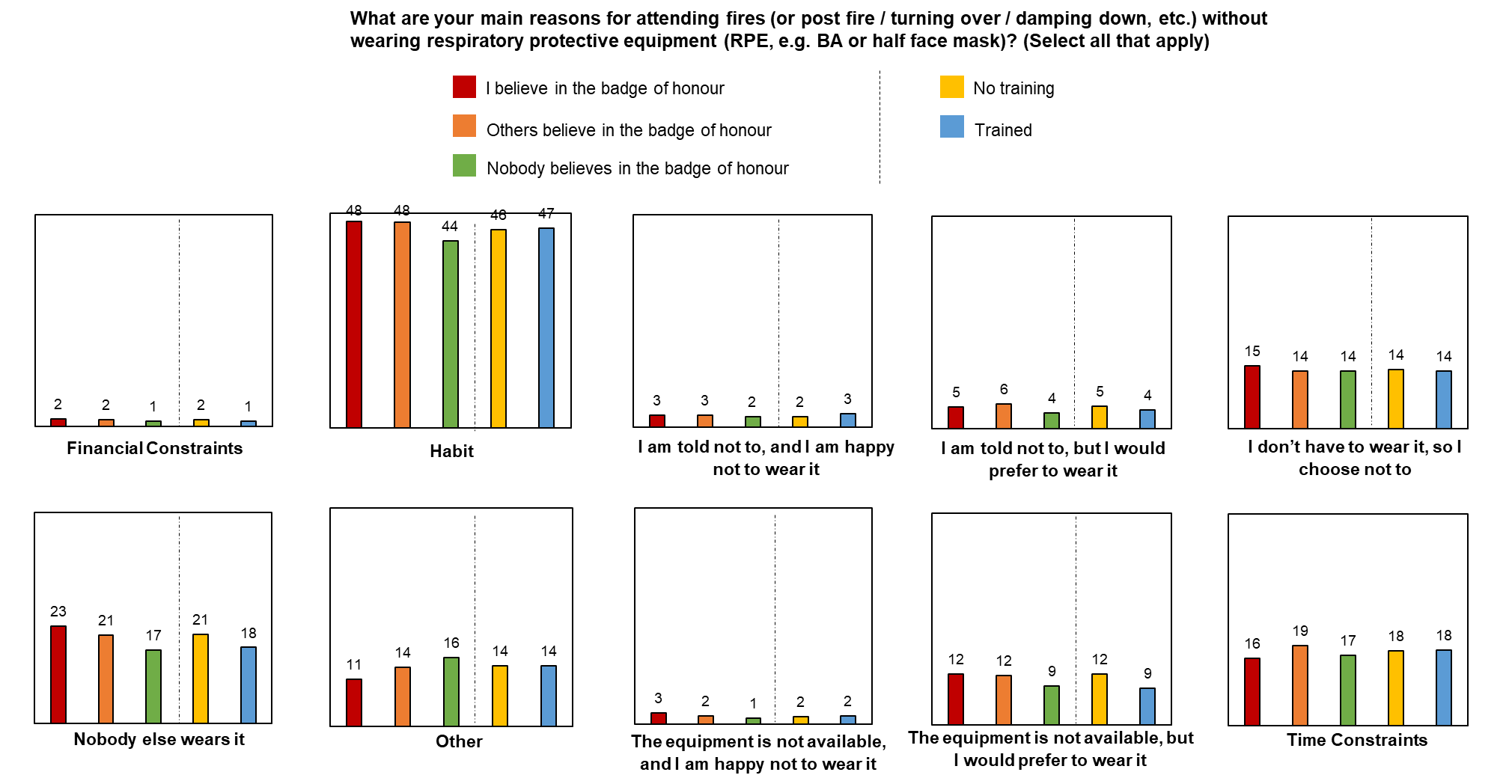


**Figure S5: Firefighters’ reasons for attending fire incidents without wearing breathing apparatus (selected from a list of options).** The percentage of firefighters from each badge of honour belief category/training status category who selected each of the listed options is presented

**Geographic Distribution of Contaminant Control Measures**

Variation among participants from the same FRS were found for all variables assessed in the survey. Two illustrative examples of this variation are provided below (Figure S6+S7), where it can be seen that participants from the same geographic regions report differing existence of/adherence to contaminant control measures.

**Figure S6**: Geographic and inter-FRS variation in the use of clean/dirty designated areas within UK fire stations.

**Figure S7**: Geographic and inter-FRS variation in whether clean and dirty PPE is stored separately within UK FRS stations.
